# Promoting Protection Against a Threat That Evokes Positive Affect: The Case of Heat Waves in the United Kingdom

**DOI:** 10.1037/xap0000083

**Published:** 2016-06-06

**Authors:** Wändi Bruine de Bruin, Carmen E. Lefevre, Andrea L. Taylor, Suraje Dessai, Baruch Fischhoff, Sari Kovats

**Affiliations:** 1 Centre for Decision Research, Leeds University Business School, University of Leeds and Department of Engineering and Public Policy, Carnegie Mellon University; 2 Centre for Decision Research, Leeds University Business School, University of Leeds and Centre for Behaviour Change, Department of Clinical, Educational and Health Psychology, University College London; 3 Centre for Decision Research, Leeds University Business School and Sustainability Research Institute, School of Earth and Environment, University of Leeds; 4 Sustainability Research Institute and ESRC Centre for Climate Change Economics and Policy, School of Earth and Environment, University of Leeds; 5 Department of Engineering and Public Policy and Department of Social and Decision Sciences, Carnegie Mellon University; 6 NIHR Health Protection Research Unit in Environmental Change and Health, London School of Hygiene and Tropical Medicine

**Keywords:** emotions, weather, heat wave, affect heuristic, availability heuristic

## Abstract

Heat waves can cause death, illness, and discomfort, and are expected to become more frequent as a result of climate change. Yet, United Kingdom residents have positive feelings about hot summers that may undermine their willingness to protect themselves against heat. We randomly assigned United Kingdom participants to 1 of 3 intervention strategies intended to promote heat protection, or to a control group. The first strategy aimed to build on the availability heuristic by asking participants to remember high summer temperatures, but it elicited thoughts of pleasantly hot summer weather. The second strategy aimed to build on the affect heuristic by evoking negative affect about summer temperatures, but it evoked thoughts of unpleasantly cold summer weather. The third strategy combined these 2 approaches and succeeded in evoking thoughts of unpleasantly hot summer weather. Across 2 experiments, the third (combined) strategy increased participants’ expressed intentions to protect against heat compared with the control group, while performing at least as well as the 2 component strategies. We discuss implications for developing interventions about other “pleasant hazards.”

Heat waves can pose a threat to health and well-being. Daily mortality increases when the weather is hotter than a population-specific threshold (Curriero et al., 2002). Syndromic surveillance data indicate increased frequency of heat-related illness during the 2013 United Kingdom heat wave (Smith et al., 2016). The 2003 heat wave was associated with approximately 35,000 excess deaths in Western Europe, including 2,000 in England, especially among older adults over the age of 75 (Johnson et al., 2005; Robine et al., 2008). Younger adults can also suffer adverse health effects after prolonged exposure to high outdoor temperatures, especially after vigorous physical activity (Glazer, 2005; Kovats & Hajat, 2008).

In 2013, the United Kingdom experienced its third warmest July since 1910 (Met Office, 2013a). Daily maximum temperatures were higher than 28 °C (or 82 °F) on 19 consecutive days, including eight days when temperatures exceeded 30 °C (or 86 °F; Met Office, 2013a). Although 2013 brought the first United Kingdom heat wave in seven years (Met Office, 2013a) experts predict that climate change will increase the frequency, intensity, and length of heat waves (Intergovernmental Panel on Climate Change, 2013).

Recommended heat protection behaviors include avoiding the midday sun, drinking plenty of liquids, and reducing alcohol intake (Hajat, O’Connor, & Kosatsky, 2010). A few studies have suggested that United Kingdom residents may be reluctant to implement recommended heat protection behaviors. In qualitative interviews, United Kingdom residents with risk factors for adverse health effects saw heat protection recommendations as unnecessary because they did not see themselves as being at risk (Abrahamson et al., 2009; Wolf, Adger, Lorenzoni, Abrahamson, & Raine, 2010). Tourists from the United Kingdom (and other Northern countries) say that they plan to stay in the sun for many hours, even during midday, when on summer holidays (Evans, Shickle, & Morgan, 2001; Manning & Quigley, 2002). Interviews with United Kingdom migrants to Spain suggest that they are less likely than locals to follow heat protection practices (Fuller & Bulkeley, 2013).

Here, we relied on behavioral decision research to promote heat protection intentions. Behavioral decision research posits that people use heuristics to make judgments about the riskiness of events and the need for protection (Slovic, Finucane, Peters, & MacGregor, 2004; Tversky & Kahneman, 1973). We developed heat protection intervention strategies that built on two well-documented heuristics. The first aimed to invoke the *availability heuristic* (Tversky & Kahneman, 1973), by increasing the salience of experiences with hot weather. The second strategy aimed to build on the *affect heuristic* (Slovic et al., 2004), and was designed to increase negative feelings about hot weather. We tested, separately and jointly, the usefulness of invoking these heuristics for promoting heat protection behaviors.

## Increasing the Salience of Risky Events: Interventions Based on the Availability Heuristic

The availability heuristic refers to using the salience of an example event as a cue for judging the likelihood that a similar event will occur (Tversky & Kahneman, 1973). For example, people who have recent experiences with floods perceive greater flood risks, likely because floods are more vivid to them (O’Connor, Yarnal, Dow, Jocoy, & Carbone, 2005; Siegrist & Gutscher, 2006). Media coverage has also been associated with higher risk perceptions, perhaps as a result of increasing the salience of adverse events (Combs & Slovic, 1979).

However, we posit that priming hot summers may not necessarily be effective for promoting concerns about heat protection among United Kingdom residents. A qualitative interview study has suggested that the prospect of increased summer temperatures elicits positive responses among United Kingdom residents, including those who may be more vulnerable to adverse health effects from heat (Abrahamson et al., 2009; Wolf et al., 2010). Indeed, correlational evidence suggests that feeling positive about heat is related to lower willingness to protect against heat (Lefevre et al., 2015). These results are consistent with other correlational evidence suggesting that people perceive less risk when they feel good about a hazard—a pattern seen for sun exposure (Bränström, Brandberg, Holm, Sjöberg, & Ullén, 2001), wood-burning fireplaces (Hine, Marks, Nachreiner, Gifford, & Heath, 2007), and speeding (Rhodes & Pivik, 2011).

## Eliciting Negative Feelings About Risky Events: Interventions Based on the Affect Heuristic

Research on the affect heuristic finds that negative affect is associated with higher perceptions of risk (Slovic et al., 2004). For example, exposure to media reports that elicit negative emotions increases perceptions of risk (Holman, Garfin, & Silver, 2014; Johnson & Tversky, 1983). Promoting fear also increases perceptions of risk and willingness to take protective action (Lerner, Gonzalez, Small, & Fischhoff, 2003; Roeser, 2012). Indeed, fear appeals may lead to behavior change, as long as people know the recommended protection strategies and recognize them as effective (Ruiter, Abraham, & Kok, 2001; Witte & Allen, 2000). In qualitative interviews, United Kingdom residents have demonstrated knowledge of recommended heat protection behaviors, including drinking plenty of water, staying out of the sun, and delaying physical activities to a cooler time of day—but they showed little motivation to implement those behaviors due to not perceiving themselves at risk (Wolf et al., 2010).

Even mild negative emotions, such as feelings of unpleasantness, can influence risk perceptions (Alhakami & Slovic, 1994; Finucane, Alhakami, Slovic, & Johnson, 2000). For example, negative mood has been linked to gray weather and lower temperatures, especially among people living in cooler climates (Keller et al., 2005; Schwarz & Clore, 1996). Negative mood also increases the likelihood of remembering unpleasant experiences in different domains, including weather (Mayer, McCormick, & Strong, 1995). When asked to provide “a description of the weather that starts with the letter c,” people in a negative mood are more likely than people in a positive mood to think of cold and cloudy weather, rather than clear and calm weather (Mayer et al., 1995).

However, we posit that priming negative emotions about events that are inherently seen as positive may not be effective for promoting risk protection behaviors. Qualitative studies have suggested that people in the United Kingdom may feel positive about hot summer weather, with negative feelings being directed at low summer temperatures (Fuller & Bulkeley, 2013; Harley, 2003; Wolf et al., 2010). If so, then efforts to evoke negative affect about summer weather in the United Kingdom will increase the salience of unpleasantly cold summers, and be relatively ineffective for promoting heat protection behaviors.

## Increasing Salience and Negative Affect: Interventions Based on the Availability *and* the Affect Heuristic

Here, we examine the joint as well as the individual effects of strategies building on the availability and the affect heuristic. That is, we examine United Kingdom residents’ responses to strategies increasing the availability of experienced hot weather, positive affect about hot weather, or both. We posit that, for focal events that are perceived as positive, warnings that aim to promote risk protection will be more effective if they build on both the availability heuristic and the affect heuristic, rather than on either heuristic alone. Specifically, increasing the availability of hot weather should trigger positive affect unless negative affect with respect to hot weather is explicitly evoked. Conversely, priming negative affect should increase recollections of cold summer weather, unless the availability of hot summer weather is explicitly evoked. Although high and unpleasant temperatures may not be salient to United Kingdom residents, qualitative interviews suggest that United Kingdom residents sometimes do complain when the weather gets hot (Harley, 2003; Wolf et al., 2010).

## Hypotheses

We report on two experiments that manipulated availability and negative affect, separately and jointly. We measured effects on recalled temperatures, pleasantness ratings of those recalled temperatures, and intentions to protect against heat. Experiment 1 was conducted after the 2013 heat wave and Experiment 2 during a hot spell in the summer of 2014. Each experiment included four groups of participants. The first group received instructions based on the availability heuristic, asking them to recall the highest temperature experienced during the past summer. The second group received instructions based on the affect heuristic, asking them to recall the most unpleasant temperature experienced in the past summer. The third group received combined instructions, asking them to recall the most unpleasant highest temperature experienced in the past summer. The control group engaged in free recall, with control participants in Experiment 1 receiving no temperature recall instructions, and control participants in Experiment 2 receiving instructions to think about “any” temperatures experienced in the past summer. All groups then reported their intentions to protect against heat. We tested the following hypotheses:

*Hypothesis 1:* Reported intentions to protect against heat are greatest among participants who are instructed to recall the “most unpleasant highest temperature,” as compared with participants in the other groups;

*Hypothesis 2:* (a) Effects of the “most unpleasant highest” versus “highest” temperature recall instructions on reported intentions to protect against heat are partially mediated by reported pleasantness. (b) Effects of the “most unpleasant highest” versus the “most unpleasant” temperature recall instructions are partially mediated by the magnitude of recalled temperatures.

## Experiment 1

### Method

#### Sample

Experiment 1 was completed by a national sample of 1,497 United Kingdom participants. Participants’ average reported age was 54.4 years (*SD* = 17.8), with 54.2% female, 94.7% White, and 44.3% with education beyond high school.

#### Procedure

Invitations to an online survey about “weather” were emailed through a professional agency (www.researchnow.co.uk). The survey was online 26–31 October 2013, three months after the hottest United Kingdom summer since 2006 (Met Office, 2013b). The mean United Kingdom-wide July temperature was 17.0 °C (range from 2.0 to 33.5 °C), which exceeded the long-term 1981–2010 average by 1.9 °C (Met Office, 2013b). In October, the mean United Kingdom-wide temperature was 11.2 °C (range from −3.6° to 23.0 °C), which registered as 1.7 °C above the long-term 1981–2010 average (Met Office, 2013c).

Participants were randomly assigned to one of four groups. The *highest temperature recall group* was asked to remember “the highest maximum temperature” they had experienced in the summer of 2013, because the availability heuristic posits that accessing past experiences will make an event seem more risky. The *most unpleasant temperature recall group* was asked to remember “the most unpleasant temperature” they had experienced in the summer of 2013, because the affect heuristic posits that evoking negative affect will make an event seem more risky. The most unpleasant highest temperature recall group received instructions that combined those from the previous two groups, and was asked to remember “the most unpleasant highest maximum temperature” they experienced in the summer of 2013. A fourth *no-instruction control group* received no temperature recall instructions.

With the exception of those in the control group, participants first reported the magnitude of the temperature they had in mind (in Celsius or Fahrenheit). These reported temperatures were transformed to Celsius for the present analyses.^1^ The pleasantness of the recalled temperature was elicited with the question, “How did you find this temperature at the time?,” which was accompanied by a response scale ranging from 1 (*very unpleasant*) to 5 (*very pleasant*).

Next, participants in all four groups rated 10 heat protection behaviors on a 5-point scale (1 = *never*; 5 = *always*). Specifically, they were asked “Next summer, during very hot days, how often would you [. . .] to protect yourself from heat?” The full question was repeated for (a) keep out of the sun between 11.00 a.m. and 3.00 p.m., (b) walk in the shade; (c) apply sunscreen, (d) avoid extreme physical exertion, (e) have plenty of cold drinks, (f) avoid excess alcohol, (g) keep windows that are exposed to sun closed during the day, (h) open windows at night when the temperature has dropped, (i) close curtains of windows that receive morning or afternoon sun, and (j) use electric fans.^2^ All heat protection behaviors, except for sunscreen application, were taken from the Heat Wave Plan for England as released by the National Health Service, Public Health England, and the Met Office (2013). The reliability of the 10 ratings was sufficient to warrant the computation of an overall averaged rating of heat protection intentions (Cronbach’s alpha = .75).

#### Analysis plan

As manipulation checks, we conducted two separate analyses of variance (ANOVAs) examining the effect of temperature recall instructions (highest, most unpleasant, or most unpleasant highest) on recalled pleasantness and temperatures for the most recent summer. The no-instruction control group was not included in these analyses, because control participants in Experiment 1 were not asked to recall or rate any temperatures. To test whether the strategy based on the availability heuristic led to recalling relatively pleasant temperatures, the first set of planned contrasts tested whether the highest temperature recall group remembered more pleasant temperatures than did those who were asked to recall the most unpleasant or the most unpleasant highest temperatures. To test whether the strategy based on the affect heuristic led to recalling relatively cool temperatures, the second set of planned contrasts tested whether reported temperatures were lower for the most unpleasant temperature recall group than for the highest and most unpleasant highest temperature recall group.

Next, we used an ANOVA to examine the effect of temperature recall instructions (highest, most unpleasant, most unpleasant highest, or control) on expressed intentions to protect against heat. To test *H*_1_, planned contrasts compared the most unpleasant highest temperature recall group against each other group. Post hoc analyses additionally compared the highest and most unpleasant temperature recall groups with the control group. To test *H*_2_, we conducted linear regressions and bootstrapping mediation tests examining whether remembered pleasantness and magnitude of temperatures statistically explained any observed effects of temperature recall instructions on heat protection intentions (Preacher & Hayes, 2008)^3^ For these tests of *H*_2_, experimental conditions were coded into dummy variables, with the highest most unpleasant temperature recall group as the reference category. They included demographic variables (e.g., age, gender, education, and race), but excluding them produced similar conclusions. For all analyses, we set α = .05 (two-sided).

### Results

#### Manipulation checks

**Figure 1 fig1:**
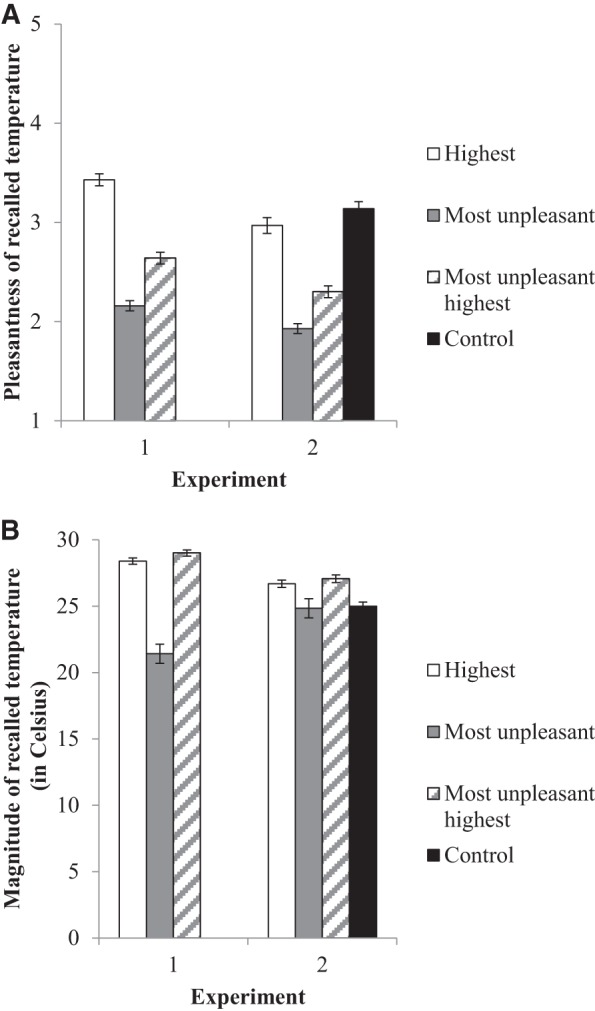
Effect of temperature recall instructions on mean (A) pleasantness ratings of recalled temperatures, and (B) magnitude of recalled temperatures. The control group in Experiment 1 recalled no temperatures, while the control group in Experiments 2 recalled “any” temperature. Error bars reflect standard errors.

Figure 1A shows mean pleasantness ratings, for the three temperature recall groups. There was a significant main effect, *F*(2, 1070) = 122.39, η^2^ = .19, *p* < .001. The planned contrasts showed that participants instructed to recall the highest temperature gave higher pleasantness ratings than did those instructed to recall the most unpleasant or the most unpleasant highest temperature (each *p* < .001). Thus, the strategy based on the availability heuristic produced relatively pleasant feelings about recalled temperatures.[Fig-anchor fig1]

Figure 1B shows the significant effect of the temperature recall instructions on mean reported temperatures in degrees Celsius, *F*(2, 1069) = 87.28, η^2^ = .14, *p* < .001. The planned contrasts showed that the reported magnitude of recalled temperatures was lower for participants who were asked to remember the most unpleasant than for those asked to remember the highest or the most unpleasant highest temperature they experienced during the past summer (each *p* < .001). Thus, the strategy based on the affect heuristic evoked recollections of relatively cooler summer temperatures.

#### Effect of temperature recall instructions on heat protection intentions (***H***_**1**_**)**

**Figure 2 fig2:**
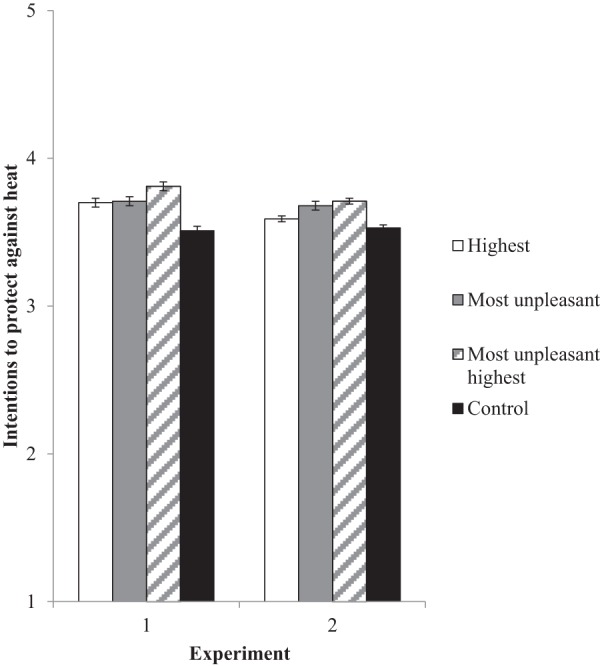
Effect of temperature recall instructions on mean intentions to protect against heat. Error bars reflect standard errors.

Figure 2 shows the statistically significant effect of temperature recall instructions on intentions to protect against heat, *F*(3, 1493) = 19.00, η^2^ = .04, *p* < .001. Planned contrasts supported *H*_1_, such that instructions to recall the most unpleasant highest temperature produced the highest level of reported heat protection intentions (vs. highest, *p* = .01; vs. most unpleasant, *p* = .02; vs. control, *p* < .001). Thus, the strategy combining the availability heuristic and the affect heuristic was more effective than invoking either separately. All three temperature-recall groups reported greater heat protection intentions than did the no-instruction control group (post hoc comparisons, each *p* < .001).[Fig-anchor fig2]

#### Role of remembered temperatures and pleasantness (***H***_**2**_**)**

Table 1 shows regression models predicting reported heat protection intentions. The significant positive effects of the most unpleasant highest temperature recall instructions (vs. each of the other conditions) on reported heat protection intentions held when including demographic variables, with older adults and women expressing greater intentions (Experiment 1, Model 1a).^4^ These effects of temperature recall instructions were no longer significant after taking into account the recalled pleasantness and magnitude of temperatures (Experiment 1, Model 1b vs. Model 2). Control participants were excluded from that analysis because, as noted, they received no temperature recall instructions.[Table-anchor tbl1]

**Figure 3 fig3:**
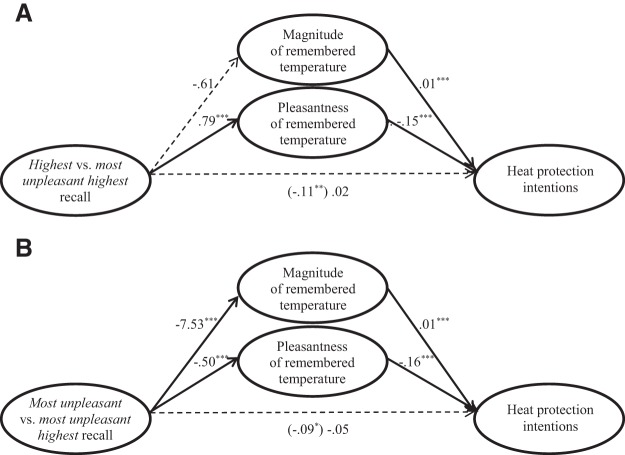
Multimediation models for effects of Experiment 1’s temperature recall instructions on intentions to protect against heat, comparing instructions to recall (A) the “highest” temperature, and (B) the “most unpleasant” temperature with instructions to recall the “most unpleasant highest” temperature. Solid lines reflect significant paths. * *p* < .01. *** *p* < .001.

Next, we conducted two mediation tests to examine whether the reported effects of temperature recall instructions on heat protection intentions were due to changes in the pleasantness and magnitude of recalled temperatures (as described in the analysis plan.) Support for *H*_2a_ is provided by the first mediation test (Figure 3A). As expected, the highest temperature recall instructions produced lower heat protection intentions than did the most unpleasant highest temperature recall instructions, due to increasing perceived pleasantness (95% CI [−.16, −.09]) for recalled temperatures that were similarly high (95% CI [−.02, .00]). Thus, the strategy based on both heuristics may have led to greater heat protection intentions than the strategy based on the availability heuristic alone because it elicited thoughts of hot weather as relatively unpleasant. Support for *H*_2b_ is provided by the second mediation test (Figure 3B), which showed that instructions to recall the most unpleasant temperature produced lower intentions for heat protection than did instructions to recall the most unpleasant highest temperature, due to recalling lower temperatures (95% CI [−.15, −.08]), despite also evoking less pleasantness (95% CI [.05, .12]). Thus, the strategy based on both heuristics may have led to greater heat protection intentions than the strategy based on the affect heuristic alone, in part, because it elicited thoughts of unpleasant weather that was relatively hot.[Fig-anchor fig3]

### Discussion

Experiment 1 found the greatest heat protection intentions among participants who were asked to recall the most unpleasant highest temperature (*H*_1_). Thus, evoking both the availability heuristic and the affect heuristic was more effective that evoking either separately. A first mediation analysis suggested that the most unpleasant highest temperature recall group had greater heat protection intentions than the highest temperature recall group because they recalled high temperatures that were less pleasant (*H*_2a_). A second mediation analysis suggested that the most unpleasant highest temperature recall group had greater heat protection intentions than the most unpleasant temperature recall group, in part, because they recalled unpleasant temperatures that were higher (*H*_2b_).

One limitation of Experiment 1 is that the control group was not asked to remember the pleasantness or magnitude of the temperatures they experienced in the most recent summer. It therefore remains unclear how much the different recall instructions influenced recalled temperatures and their rated pleasantness as compared with any memories control participants may have had, and how those changes may have contributed to heat protection intentions. Control participants in Experiment 2 were therefore asked to think of any temperature they experienced, and to indicate their magnitude and pleasantness, in addition to reporting heat protection intentions.

A second limitation of Experiment 1 is that participants received the temperature recall instructions only once. However, most public warnings are repeated, which increases their perceived credibility (Schwarz, Sanna, Skurnik, & Yoon, 2007). As a result, Experiment 2 compared responses of participants who had been in Experiment 1 with participants who received the manipulation for the first time.

## Experiment 2

### Method

#### Sample

Experiment 2 was conducted with two national samples from the United Kingdom. First, the *repeat sample* included participants who had previously completed Experiment 1.^5^ In total, 789 of the 1,497 participants returned for Experiment 2 after previously completing Experiment 1 (i.e., 52.7%). Their average age was 57.2 years (*SD* = 17.1), with 49.1% female, 94.7% White, and 42.2% reporting education beyond high school. Experiment 1 participants who returned for Experiment 2 were not significantly different from those who did not, in terms of gender, χ(1) = 1.60, *p* = .21, ethnicity, χ(1) = .56, *p* = .45, education, χ(1) = 1.52, *p* = .22, or the experimental condition to which they had been assigned in Experiment 1, χ(3) = 1.39, *p* = .71. Nor were they different in terms of the heat protection intentions they had reported in Experiment 1, *t*(1495) = −1.41, *p* = .16.^6^ The only significant difference was that those who returned for Experiment 2 were significantly older than those who did not (*M* = 57.2, *SD* = 17.7 vs. *M* = 49.1, *SD* = 19.7), *t*(1493) = −8.55, *p* < .001. As in Experiment 1, we present regression analyses assessing the effect of temperature recall instructions on intended heat protection behaviors, while controlling for age and other demographic variables (see Table 1).

Second, we also recruited a *new sample* of participants who had not previously participated in our temperature recall experiments (*n* = 1,062). On average, they were 55.4 (*SD* = 16.7) years old, with a total of 57.8% being female, 95.0% being White, 44.5% having received education beyond high school. Compared with the repeat sample, the new sample was older, *t*(2325) = 2.44, *p* < .001, and involved more women, χ(1) = 15.72, *p* < .001, but was not significantly different in terms of their race χ(1) = .11, *p* = .74, having completed education beyond high school, χ(1) = 1.03, *p* = .31, or assigned temperature recall instructions, χ(3) = 1.62, *p* = .66. Analyses that compared the new sample with the repeat sample controlled for age and gender.

#### Procedure

Experiment 2 was conducted during a hot spell in July 2014. The mean United Kingdom temperature for that month was 16.3 °C (range from 0.8 °C to 32.3 °C), which exceeded the long-term 1981–2010 average by 1.2 °C (Met Office, 2014). The survey was online 18–29 July 2014.

To recruit participants for Experiment 2’s new sample, we used the same procedure as in Experiment 1. Thus, the new sample and the repeat sample were recruited in the same way. The new participants were randomly assigned to the highest temperature recall group, the most unpleasant temperature recall group, the most unpleasant highest temperature recall group, or the control group. Repeat participants remained in the same group to which they had been assigned in Experiment 1.

Experiment 2 followed the same procedure as Experiment 1, with three exceptions. First, temperature recall instructions referred to the summers of 2013 and 2014 rather than just the summer of 2013. Second, the control group was asked to recall any temperature experienced in the summers of 2013 and 2014, including those who had been in Experiment 1’s no-instruction control group. Third, the questions about the 10 heat protection intentions were asked about the current summer rather than the next one (i.e., ‘This summer, during very hot days, how often would you [. . .] to protect yourself from heat?) Reliability of reported heat protection intentions was sufficient to warrant averaging across the 10 ratings (Cronbach’s alpha = .72).

#### Analysis plan

The analysis plan for Experiment 2 was the same as the analysis plan for Experiment 1, with two exceptions. First, the reported ANOVAs examined the effect of sample (repeat vs. new) in addition to temperature recall instructions (most unpleasant highest vs. highest, most unpleasant, or any) on variables of interest, while taking into account sample differences in age and gender. Second, the control group was included in analyses involving the pleasantness and magnitude of remembered temperatures. As noted, this change was possible because control participants in Experiment 2 were asked to recall the pleasantness and magnitude of any recent summer temperature experienced while in Experiment 1 they received no recall instructions.

### Results

#### Manipulation checks

Figure 1A shows the mean pleasantness of remembered summer weather, for participants receiving the different temperature recall instructions. We found a significant effect of temperature recall instructions on these pleasantness ratings, *F*(3, 2282) = 117.48, η^2^ = .13, *p* < .001. As in Experiment 1, the planned contrasts showed significantly higher pleasantness ratings among participants who recalled the highest rather than the most unpleasant or the most unpleasant highest temperature (each *p* < .001). Thus, the strategy based on the availability heuristic evoked recollections of relatively pleasant summer temperatures. However, the highest temperature recall group reported less pleasant temperatures than did the any temperature control group (*p* < .01). The repeat and new sample were relatively similar in pleasantness ratings, *F*(1, 2282) = 2.89, η^2^ = .00, *p* = .09. There was no significant interaction between sample type and temperature recall instructions, *F*(3, 2282) = 2.41, η^2^ = .00, *p* = .07.

Figure 1B shows the mean recalled temperatures (in degrees Celsius), as reported after receiving the different temperature recall instructions. There was a significant main effect of temperature recall instructions, *F*(3, 2246) = 10.38, η^2^ = .01, *p* < .001. As in Experiment 1, the planned contrasts showed significantly lower remembered temperatures in response to instructions to recall the most unpleasant rather than the highest or the most unpleasant highest temperature (each *p* < .001). Thus, the strategy based on the affect heuristic produced reports of relatively cool summer temperatures. There was no significant difference in recalled temperatures between the most unpleasant and any temperature recall groups (*p* = .94). The repeat sample recalled significantly higher temperatures than did the new sample (*M* = 26.88, *SD* = 7.27 vs. *M* = 25.35, *SD* = 8.60), *F*(1, 2246) = 16.75, η^2^ = .01, *p* = .01. However, there was no significant interaction, suggesting that the effect of temperature recall instructions was relatively similar in the two samples, *F*(1, 2246) = 2.26, η^2^ = .00, *p* = .08.

#### Effect of temperature recall instructions on heat protection intentions (***H***_**1**_**)**

Figure 2 shows the effect of temperature recall instructions on intentions to protect against heat. We found a significant effect of temperature recall instructions on heat protection intentions, *F*(3, 2274) = 10.70, η^2^ = .01, *p* < .001. Partial support was found for *H*_1_ in planned contrasts that compared the most unpleasant highest temperature recall group with the other three groups. Specifically, the most unpleasant highest temperature recall group had significantly greater heat protection intentions than the control group (*p* < .001) and the highest temperature recall group (*p* = .01), with the latter two being similar to each other (*p* = .06 for post hoc comparison). There was no significant difference in heat protection intentions between the most unpleasant highest temperature recall group and the most unpleasant temperature recall group (*p* = .74), which also had significantly greater heat protection intentions than the control group (*p* < .001 for post hoc comparison). Thus, the combined strategy that was based on the availability heuristic and the affect heuristic was more effective than the strategy based on the availability heuristic alone, and as effective as the strategy based on the affect heuristic alone.

Although the repeat sample expressed higher heat protection intentions than the new sample (*M* = 3.70, *SD* = .59 vs. *M* = 3.58, *SD* = .59), *F*(1, 2274) = 25.95, η^2^ = .01, *p* < .001, the effect of temperature recall instructions did not differ significantly between the two samples, *F*(3, 2274) = .29, η^2^ = .00, *p* = .83.

#### Role of remembered temperatures and pleasantness (***H***_**2**_**)**

Table 1 shows regression models predicting reported heat protection intentions. The significant effects of most unpleasant highest (vs. control and highest) temperature recall instructions remained after including demographic variables, with older adults and women expressing greater intentions (Experiment 2, Model 1). These effects of the most unpleasant highest temperature recall instructions were no longer significant after taking into account the recalled pleasantness and magnitude of temperatures (Experiment 2, Model 2).

**Figure 4 fig4:**
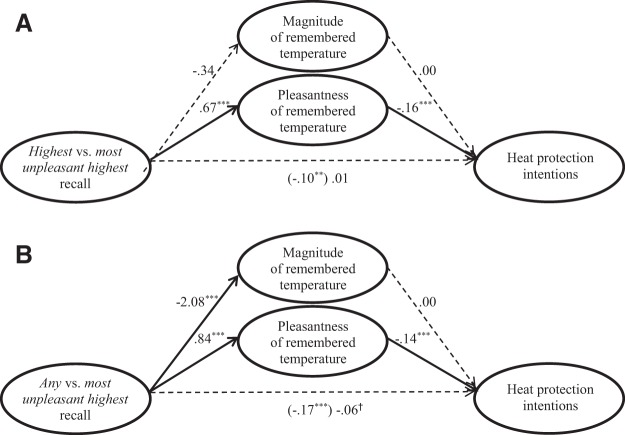
Multimediation models for effects of Experiment 2’s temperature recall instructions on intentions to protect against heat, comparing instructions to recall (A) the “highest” temperature, and (B) “any” temperature (control group) with instructions to recall the “most unpleasant highest” temperature. Solid lines reflect significant paths. ^†^ *p* < .05. *** *p* < .001.

Next, we conducted mediation tests to examine whether reported pleasantness and magnitude of recalled temperatures contributed to the reported effectiveness of the most unpleasant highest temperature recall instructions. Support for *H*_2a_ is seen in the first mediation test (Figure 4A), which found that instructions to recall the highest temperatures produced lower heat protection intentions than did instructions to recall the most unpleasant highest temperatures, due to participants recalling more pleasant temperatures (95% CI [−.14, −.08]) while recalling similar magnitudes (95% CI [−.01, .01]). Thus, as in Experiment 1, the strategy that combined the availability and the affect heuristic may have led to greater heat protection intentions than the strategy based on the availability heuristic alone, in part, because it elicited thoughts of hot weather that was relatively unpleasant. Unlike Experiment 1, however, we conducted no mediation test for *H*_2b_ because tests for *H*_1_ showed no significant difference between the most unpleasant highest and the highest recalled temperatures.[Fig-anchor fig4]

We did conduct another mediation test (Figure 4B) to examine why instructions to recall the control group’s instructions to recall any temperature may have produced lower intentions for heat protection than did instructions to recall the most unpleasant highest temperature. We found that the effect was statistically accounted for by the any temperature recall instructions leading to reports of more pleasant temperatures as compared with the most unpleasant highest temperature recall instructions (95% CI [−.15, −.09]) despite recalling similar magnitudes (95% CI [.00, .01]). Both mediation patterns were similar in the repeat and the new sample.

### Discussion

Experiment 2 partly replicated the findings from Experiment 1, with the overall pattern of findings suggesting that reducing positive affect about heat can improve United Kingdom residents’ intentions to protect against heat. As in Experiment 1, we found that the most unpleasant highest temperature recall instructions was among the most effective in promoting heat protection intentions. It led to greater heat protection intentions than the highest and any temperature recall instructions, possibly due to eliciting thoughts of hot weather that was relatively unpleasant. It was as good as the most unpleasant temperature recall instructions, which, compared with Experiment 1, elicited thoughts of relatively high temperatures.

In Experiment 1, the greater heat protection intentions in the most unpleasant highest (as compared with the most unpleasant) temperature recall conditions had been partially due to participants reporting higher temperatures (Figure 3B). In Experiment 2, the most unpleasant highest (vs. most unpleasant) temperature recall instructions also led participants to recall higher temperatures, but the significant differences were less pronounced than in Experiment 1 (see Figure 1).^7^ Although we do not know why these differences between experiments occurred, it is possible that they reflect the differential weather conditions experienced during Experiment 1 versus Experiment 2. Experiment 1 was conducted 3 months after the 2013 heat wave, when temperatures had already been cooling down and remembered high temperatures may have seemed especially pleasant. Experiment 2 was conducted during a period of very hot weather, when positive affect about high temperatures may have been somewhat lower. Under the latter conditions, it might be easier to recall more unpleasant hot weather even when it is not explicitly mentioned in temperature recall instructions. Thus, recent weather and time of year may play an important role in motivating heat protection behaviors. Yet, across the weather conditions for the reported experiments, the main conclusion holds that the most unpleasant highest temperature recall instructions were consistently better than or as good as the alternative strategies for promoting heat protection intentions.

Unlike Experiment 1, the control group in Experiment 2 did receive temperature recall instructions, which asked them to report the magnitude and pleasantness of any recalled temperatures. This control group recalled temperatures that were moderately high for the United Kingdom (25 °C) and pleasant—as compared with the other groups in Experiments 1 and 2. Experiment 2 was conducted during a period of hot weather, making moderately high and pleasant temperatures easy to come to mind. It is possible that the control group would have recalled different temperatures if the study had been conducted at a different time of year, or during different types of weather. Although we cannot know what temperatures Experiment 1’s control group participants had in mind, like Experiment 2’s control group they had lower intentions to protect against heat, as compared with the other temperature recall groups.

Experiment 2 included two subsamples, including a returning sample that had already participated in Experiment 1, as well as a new sample recruited just for Experiment 2. In both samples, the overall pattern of results was similar. However, the repeat sample reported greater heat protection intentions than the new sample in Experiment 2, perhaps reflecting their repeated exposure to our experimental procedures (as in Schwarz et al., 2007). It is unlikely that these results reflect repeat participants’ greater inherent (prerecruitment) interest in heat protection, because in Experiment 1 they reported similar heat protection intentions as those who did not return for Experiment 2. Thus, messages that aim to motive people to protect against heat may need to repeat primes of unpleasant hot weather over time because in our experiments it was consistently among the most effective.

## General Discussion

Heat waves can cause death, illness, and discomfort, and are expected to become more frequent as a result of climate change. Yet, United Kingdom residents seem to think of hot summers with fondness (Abrahamson et al., 2009; Wolf et al., 2010). Correlational findings have suggested that positive affect about heat is related to lower heat protection intentions among United Kingdom residents (Lefevre et al., 2015).

Here, we relied on behavioral decision research to promote heat protection intentions. Behavioral decision research suggests that the perceived need for risk protection may be judged through the availability or the affect heuristic (Slovic et al., 2004; Tversky & Kahneman, 1973). We therefore developed a strategy that aimed to invoke the availability heuristic by asking participants to remember high summer temperatures. However, it elicited thoughts of pleasantly hot summer weather. Our second strategy aimed to build on the affect heuristic by priming negative affect about summer temperatures, but it evoked thoughts of unpleasantly cold summer weather. Our third strategy combined these two approaches and succeeded in eliciting thoughts of unpleasant hot summer weather. Across two experiments, the third (combined) strategy increased participants’ expressed intentions to protect against heat compared with the control group, while performing at least as well as the two component strategies. The relative effectiveness may partly depend on the weather and the time of year, with reminders of the unpleasantness of hot weather seeming more important when United Kingdom weather has recently been cooler (and hot weather may be wished for.)

One limitation of the reported research is its focus on United Kingdom residents, who may be unusual in their positive affect about heat. Indeed, hot summer weather may be associated with negative affect among residents of the southern areas of the United States and Europe (Keller et al., 2005). However, the overall pattern of results might generalize to other hazards that evoke positive affect in some populations, such as sun bathing (Bränström et al., 2001), wood-burning fire places (Hine, Marks, Nachreiner, Gifford, & Heath, 2007), and speeding (Rhodes & Pivik, 2011). In such cases, interventions may be more effective if they combine insights from the availability and affect heuristics, rather than building on either heuristic alone.

A second limitation is that we relied on participants’ self-reported heat protection intentions, rather than observations of their actual behavior. Although our temperature recall instructions did affect self-reported heat protection intentions, conclusions about effects on actual behavior would be premature.^8^ Our paper does provide the initial evidence that may be needed to convince health agencies to vary the wording of their messages, when aiming to promote heat protection behaviors. Follow-up work should examine effects on people’s actual heat protection behaviors or their experienced health effects from heat.

A third limitation is that the effects of intervention strategies were relatively small. Yet, interventions with small effect sizes can be clinically significant when taking into account the size of the target population (Rosenthal, 1990). Well-designed national campaigns could possibly amplify our findings. It would be relatively straightforward to add temperature recall primes to existing heat protection warnings in the United Kingdom. Ready platforms include the heat protection warnings released by the National Health Service, Public Health England, and the Met Office (2013).

Those messages could, of course, also incorporate other theoretically-grounded approaches for promoting heat protection. Strategies that have been effective in other contexts include emphasizing social norms to protect against risk (Schultz, Nolan, Cialdini, Goldstein, & Griskevicius, 2007), and the regret that may be felt after engaging in risky behaviors (Richard, van der Pligt, & De Vries, 1996). Moreover, it has been shown that emotion-based appeals may not be effective if they leave people uninformed about what to do (Ruiter et al., 2001). United Kingdom residents can name heat protection behaviors (Wolf et al., 2010) but it is possible that they have incomplete mental models about how to effectively implement those behaviors (Morgan et al., 2002). Content should be designed to target the communication needs of the intended audiences in understandable language and personally relevant contexts, and be tested before widespread implementation (Bruine de Bruin & Bostrom, 2013; Fischhoff, Brewer, & Downs, 2011; Noar, Benac, & Harris, 2007). Long-term mass campaigns in Australia have suggested that sun-protective behaviors are amenable to change, across age groups and segments of the population (Dobbinson et al., 2008). Thus, there is promise for promoting United Kingdom residents’ safe enjoyment of hot summer weather.

## Supplementary Material

10.1037/xap0000083.supp

## Figures and Tables

**Table 1 tbl1:** Regression Analyses (Unstandardized B) Predicting Intentions to Protect Against Heat

Variable	Experiment 1			Experiment 2	
	Model 1a	Model 1b	Model 2	Model 1	Model 2
Control vs. most unpleasant highest group	−.29***	—	—	−.18***	−.04
Highest vs. most unpleasant highest group	−.10*	−.11*	.03	−.10***	.00
Most unpleasant vs. most unpleasant highest group	−.10*	−.09*	−.06	−.01	−.06^†^
Reported pleasantness of recalled temperature	—	—	−.16***	—	−.15***
Reported magnitude of recalled temperature	—	—	.01***	—	.00*^b^
Repeat (vs. new) sample	—	—	—	.14***	.11***
Age	.00^a^***	.01***	.01***	.01***	.01***
Female	.26***	.27***	.26***	.29***	.29***
White	.00	.04	.04	−.11^†^	−.09^†^
Education beyond high school	−.04	−.04	−.03	−.03	−.03
*R*^2^	.12	.10	.23	.10	.18
*F*-test of model change	*F*(7, 1474) = 27.48***	*F*(6, 1048) = 18.50***	*F*(2, 1046) = 88.01***	*F*(8, 2217) = 30.04***	*F*(2, 2215) = 113.17***
*Note.* The control group in Experiment 1 recalled no temperatures, while the control group in Experiment 2 recalled “any” temperature. Interactions between age and temperature recall instructions were not significant in any of the experiments (not shown).					
^a^ Estimate (*B* = .005) is significantly different from 0. ^b^ Estimate (*B* = .003) is significantly different from 0.					
^†^ *p* < .10. * *p* < .05. *** *p* < .001.					

## Footnotes

1 All reported temperatures were transformed to the Celsius scale, after checking for anomalous responses that appeared to have been given in the wrong unit of measurement (e.g., reporting 70 °C rather than °F). Specifically, participants who had indicated that they were using the Fahrenheit scale but gave responses that would have ended up below the all-time minimum temperature recorded in the United Kingdom between May and September (−9.4 °C), were treated as having used the Celsius scale. Likewise, responses given on the Celsius response scale that were above the all-time maximum temperature recorded in the United Kingdom between May and September (38.5 °C) were treated as having used the Fahrenheit scale.

2 Participants were also asked about keeping an eye on the isolated, elderly, and ill, as well as on babies and children. These items were excluded because they were not self-protective, could not be implemented by all participants, and were not included in our previous work on the association between heat protection behavior and positive affect (Lefevre et al., 2015).

3 We used the “indirect” macro procedure for SPSS, which is available from http://www.afhayes.com/spss-sas-and-mplus-macros-and-code.html. Mediation tests were only applied to significant group differences. Mediation tests could not be applied to understand the observed differences in heat protection intentions between the most unpleasant highest temperature recall group and the no-instruction control group in Experiment 1, because the latter did not report temperatures or rate their pleasantness.

4 The effectiveness of temperature recall instructions did not vary with age (not shown), even though older age is a risk factor for experiencing adverse health effects from heat. Other studies have also found that older adults and younger adults are similarly responsive to heat protection messages, perhaps because older adults do not like to think of themselves as different from younger adults (Abrahamson et al., 2009).

5 Before Experiment 1 participants returned for Experiment 2, they received a so-called booster session in April 2014, to remind them of their temperature recall instructions before it got hot. The booster session had the same design as Experiment 2. Reported heat protection intentions were relatively similar across Experiment 2 and the booster session, *F*(1, 729) = 3.54, η^2^ = .01, *p* = .06, with relatively similar responses to the temperature recall instructions across the two, *F*(1, 729) = 2.24, η^2^ = .01, *p* = .08.

6 Across participants who completed both experiments (*n* = 789), there was no significant main effect of Experiment 1 versus 2 on heat protection intentions, *F*(1, 753) = .02, η^2^ = .00, *p* = .88. However, there was a significant interaction between experiment and temperature recall conditions on intentions to protect against heat, *F*(3, 753) = 3.32, η^2^ = .01, *p* = .02. Most notably, among these repeat participants, the difference between the most unpleasant highest and the most unpleasant temperature recall groups was only significant in Experiment 1 (*M* = 3.86, *SD* = .61 vs. *M* = 3.69, *SD* = .68; *p* = .01) and not in Experiment 2 (*M* = 3.80, *SD* = .54 vs. *M* = 3.75, *SD* = .63; *p* = .39).

7 In both experiments, differences in recalled temperatures between these conditions were larger than the just noticeable difference in indoor temperature, which presumably lies in the range of .09--.80 °C (Lee, Cho, Yun, & Lee, 1998).

8 For different research purposes (e.g., Khare et al., 2015; Lefevre et al., 2015), participants in both experiments also rated the frequency of recent heat protection behaviors. Experiment 1 asked participant to rate the frequency of heat protection behaviors “during the heat wave of 2013.” Experiment 2 asked participants to rate the frequency of heat protection behaviors “so far this summer (2014).” Experiment 1 revealed a significant effect of temperature recall instructions on the rated frequency of past behaviors, *F*(3, 1493) = 6.17, η^2^ = .01, *p* < .001, as did Experiment 2, *F*(3, 2259) = 5.26, η^2^ = .01, *p* < .001. Moreover, Experiment 2 revealed no significant interaction between sample type and temperature recall instructions, such that the repeat sample and the new sample gave reports of past behaviors that were influenced by their temperature recall instructions. Because participants of Experiment 1 and new-sample participants of Experiment 2 had not received temperature recall instructions until *after* those recent behaviors could have been implemented, we conclude that the recall of past behaviors was likely influenced by temperature recall instructions. Although this finding suggests that temperature recall instructions were effective in terms of changing participants’ motivation for heat protection, conclusions about the effect of temperature recall instructions on actual heat protection behavior should be drawn with caution. More details are provided in the supplemental materials.
