# Supplementary material for: Promoting Protection Against a Threat That Evokes Positive Affect: The Case of Heat Waves in the United Kingdom
Source: J Exp Psychol Appl. 2016 Jun 6;22(3):261–71. doi: 10.1037/xap0000083 (PMC5015596; doi:10.1037/xap0000083)
Supplement: Supplementary file 1 [file zep002162321sb01.docx]

**Supplemental Materials**

**Promoting Protection Against a Threat That Evokes Positive Affect: The Case of Heatwaves in the UK**

**by W. Bruine de Bruin et al., 2016, *JEP: Applied***

**http://dx.doi.org/10.1037/xap0000083**

**Supplemental Materials: Ratings of recent behavior**

Because our experiments were conducted as part of a larger data collection effort, we had access to measures of self-reported past behavior that were collected to answer other research questions. Those measures were collected in Experiment 1 and in Experiment 2. Below, we describe the measures, and repeat the main analyses reported in the main text of the paper, with the dependent variable reflecting ratings of recent heat protection behavior instead of heat protection intentions. Specifically, the analyses focus on the effects of temperature recall instructions as well as the underlying role of the pleasantness and magnitude recalled temperatures.

**Method**

Experiment 1 and Experiment 2 included questions that asked participants to rate the frequency of recent heat protection behaviors, on a 5-point scale (1 = never; 5 = always). Experiment 1 asked how often participants engaged in ten behaviors “during the heatwave of 2013,” and Experiment 2 asked how often participants engaged in ten behaviors “so far this summer (2014).” The rated behaviors included (1) kept out of the sun between 11:00am and 3:00pm; (2) if you had to go out in the heat, stayed in the shade; (3) if you had to go out in the heat, applied sun screen; (4) avoided extreme physical exertion; (5) had plenty of cold drinks; (6) avoided excess alcohol; (7) kept windows that were exposed to the sun closed during the day; (8) opened windows at night when the temperature had dropped; (9) closed curtains that received morning or afternoon sun; (10) used an electric fan. Reliability across the ten ratings was sufficient to warrant the computation of an averaged measure (α = .80 for Experiment 1; α = .80 for Experiment 2). Responses to the averaged ratings across the ten behaviors were significantly correlated to reported intentions (*r* = .80, *p* < .001 for Experiment 1; *r* = .82, *p* < .001 for Experiment 2).

**Results**

Effect of temperature recall instructions on ratings of recent behavior

For Experiment 1, we conducted an ANOVA to examine the effect of temperature recall instructions (highest, most unpleasant, most unpleasant highest, or control) on ratings of recent heat protection behaviors (Figure S1). We found a significant effect of temperature recall instructions, *F*(3, 1493) = 6.17, *η^2^* = .01, *p* < .001. Planned contrasts compared the ‘most unpleasant highest’ temperature recall group against each other group, and revealed that it yielded significantly higher ratings than did the ‘most unpleasant’ temperature recall group (*p* = .04) and the control group (*p* < .001). There was no significant difference between the ‘most unpleasant highest’ and ‘highest’ temperature recall groups (*p* = .17). Post-hoc analyses additionally found that the ‘highest’ temperature recall group reported more frequent recent heat protection behaviors than did the control group (*p* < .01), as did the ‘most unpleasant’ temperature recall group (*p* = .05).

For Experiment 2, we conducted an ANOVA to examine the effect of temperature recall instructions (highest, most unpleasant, most unpleasant highest, or control) on ratings of recent heat protection behaviors (Figure S1), while taking into account the sample (new vs. repeat). We found a significant effect of the temperature recall instructions, *F*(3, 2259) = 5.26, *η^2^* = .01, *p* < .001. Planned contrasts showed that the rated frequency of recent heat protection behaviors was significantly greater for the ‘most unpleasant highest’ temperature recall group than for the ‘highest’ temperature recall group (*p* = .02) and the ‘any’ temperature recall control group (*p* < .01), but showed no difference with those provided by participants in the ‘most unpleasant’ temperature recall group (*p* = .72). Post-hoc analyses further confirmed that the ‘most unpleasant’ temperate recall group reported more frequent recent heat protection behaviors than the control group (*p* < .01) but ‘highest’ temperature recallers did not (*p* = .58). Responses to temperature recall instructions did not interact with the type of sample, *F*(3, 2259) = .29, *η^2^* = .00, *p* = .83. However, the rated frequency of recent heat protection behavior was significantly higher in the repeat sample than in the new sample, *F*(1, 2259) = 9.32, *η^2^* = .01, *p* < .01.

Role of remembered temperatures and pleasantness

Table S1 shows linear regressions predicting the rated frequency of recent heat protection behaviors. After taking into account demographic variables for Experiment 1, the difference between the ‘any’ and ‘most unpleasant highest’ temperature recall groups remained significant, while the difference between the ‘most unpleasant’ and the ‘most unpleasant highest’ temperature recall groups became marginally significant (Model 1). The latter correlation was further reduced after considering that the rated frequency of past behaviors was significantly related to the reported pleasantness and magnitudes of recalled temperatures (Model 1b vs. Model 2). As in the ANOVA reported above, the difference between the ‘highest’ and the ‘most unpleasant highest’ temperature recall groups were not significant. Because the control group involved no assessment of perceived temperatures, it was not included in these analyses (Model 1b, Model 2). Figure S2 presents a mediation model to examine the process underlying the marginal difference between the ‘most unpleasant’ and ‘most unpleasant highest’ temperature recall groups, using bootstrapping mediation tests (Preacher & Hayes, 2008) including demographic variables (e.g., age, gender, education, and race). It shows significant mediation paths for both the magnitude of recalled temperatures (*95% CI* = -.15, -.06) and their rated pleasantness (*95% CI* = .03, .09).

For Experiment 2, the effects of ‘most unpleasant highest’ temperature recall instructions remained significant after taking into account demographic variables (Model 1; Table S1). Figure S3 shows the results of the associated bootstrapping mediation tests (Preacher & Hayes, 2008), while taking into account demographic variables (e.g., age, gender, education, and race) and sample differences (e.g., new vs. repeat). Figure S3A shows that the difference between the ‘highest’ and ‘most unpleasant highest’ temperature recall groups is significantly mediated by ratings of pleasantness (*95% CI* = -.13, -.07), but not by the magnitude of recalled temperatures (*95% CI* = -.01, .00). Similarly, Figure S3B shows that the difference between the ‘any’ and ‘most unpleasant highest’ temperature recall groups is significantly mediated by ratings of pleasantness (*95% CI* = -.15, -.08) but not by the magnitude of recalled temperatures (*95% CI* = -.01, .01).

Table S1: Regression analyses (unstandardized *B*) predicting ratings of recent heat protection behaviors.

|  | Experiment 1 | | |  | Experiment 2 | |
| --- | --- | --- | --- | --- | --- | --- |
|  | Model 1a | Model 1b | Model 2 |  | Model 1 | Model 2 |
| Control vs. most unpleasant highest group | -.18^***^ | - | - |  | -.15^***^ | -.02 |
| Highest vs. most unpleasant highest group | -.06 | -.06 | .04 |  | -.11^**^ | -.01 |
| Most unpleasant vs. most unpleasant highest group | -.09^+^ | -.09^+^ | -.05 |  | -.01 | -.06 |
| Reported pleasantness of  recalled temperature | - | - | -.12^***^ |  | - | -.14^***^ |
| Reported magnitude of  recalled temperature | - | - | .01^***^ |  | - | .00 |
| Repeat vs. new sample | - | - | - |  | .07^*^ | .07^*^ |
| Age | .01^***^ | .01^***^ | .01^***^ |  | .01^***^ | .01^***^ |
| Female | .31^***^ | .32^***^ | .32^***^ |  | .38^***^ | .38^***^ |
| White | .12 | .12 | .11 |  | -.01 | -.01 |
| Higher education | -.06^+^ | -.06 | -.06 |  | -.09^**^ | -.09^**^ |
| *R^2^* | .09 | .15 | .15 |  | .10 | .16 |
| *F*-test of model change | *F*(7,1074)=  21.62^***^ | *F*(6,1048)=  19.45^***^ | *F*(2,1046)=  42.62^***^ |  | *F*(8,2204)=  31.47^***^ | *F*(2,2204)=  71.64^***^ |

^+^ p < .10; ^*^ *p* < .05; ^**^ *p* < .01; ^***^ *p* < .001;

Note: The control group in Experiment 1 recalled no temperatures, while the control group in Experiments 2 recalled ‘any’ temperature. Figure S1: Effect of temperature recall instructions on mean rated frequency of recent heat protection behaviors.

Figure S2: Multi-mediation models testing effects for Experiment 1’s instructions to recall the ‘most unpleasant highest’ temperatures vs. instructions to recall the ‘most unpleasant’ temperature on rated frequency of recent heat protection behaviors.

Magnitude of remembered temperature

-7.53^***^

.01^***^

Pleasantness of remembered temperature

-.11^***^

-.50^***^

)

Rated frequency of recent heat protection

(-.09^+^) -.05

*Most unpleasant* vs. *most unpleasant highest* recall

Note: Solid lines reflect significant paths.

Figure S3: Multi-mediation models for effects of Experiment 2’s temperature recall instructions on rated frequency of recent heat protection behaviors, comparing instructions to recall the ‘most unpleasant highest’ temperatures vs. instructions to recall (A) the ‘highest’ temperature and (B) ‘any’ temperature.

Magnitude of remembered temperature

Pleasantness of remembered temperature

(A)

-.32

.00

-.15^***^

.67^***^

Rated frequency of recent heat protection

*Highest* vs. *most unpleasant highest* recall

(-.11^**^) -.01

Magnitude of remembered temperature

Pleasantness of remembered temperature

(B)

-2.07^***^

-.13^***^

.00

.84^***^

Rated frequency of recent heat protection

*Any* vs. *most unpleasant highest* recall

(-.15^***^) -.04

Note: Solid lines reflect significant paths.
